# Characteristics and intrasubject variation in the respiratory microbiome in interstitial lung disease

**DOI:** 10.1097/MD.0000000000033402

**Published:** 2022-04-07

**Authors:** Jun Yeun Cho, Mi Yeon Kim, Ji Hyoun Kim, Eung-Gook Kim, Sun-Hyung Kim, Bumhee Yang, Hyeran Kang, Ki Man Lee, Kang Hyeon Choe, Yoon Mi Shin

**Affiliations:** a Division of Pulmonary and Critical Care Medicine, Department of Internal Medicine, Chungbuk National University Hospital, Chungbuk National University College of Medicine, Cheongju, Republic of Korea; b Academic Cooperation Foundation, Chungbuk National University Industry, Cheongju, Republic of Korea; c Division of Rheumatology, Department of Internal Medicine, Chungbuk National University Hospital, Chungbuk National University College of Medicine, Cheongju, Republic of Korea; d Department of Biochemical and Medical Research Center, Chungbuk National University College of Medicine, Cheongju, Republic of Korea.

**Keywords:** bronchoalveolar lavage, dysbiosis, human microbiome, interstitial lung disease, sputum

## Abstract

Recent studies have reported that the lower airway microbiome may play an essential role in the development and progression of interstitial lung disease (ILD). The aim of the current study was to evaluate the characteristics of the respiratory microbiome and intrasubject variation in patients with ILD. Patients with ILD were recruited prospectively for 12 months. The sample size was small (n = 11) owing to delayed recruitment during the COVID-19 pandemic. All subjects were hospitalized and were evaluated by a questionnaire survey, blood sampling, pulmonary function test, and bronchoscopy. Bronchoalveolar lavage fluid (BALF) was obtained at 2 sites, the most and least disease-affected lesions. Sputum collection was also performed. Furthermore, 16S ribosomal RNA gene sequencing was performed using the Illumina platform and indexes of α- and β-diversity were evaluated. Species diversity and richness tended to be lower in the most-affected lesion than in the least-affected lesion. However, taxonomic abundance patterns were similar in these 2 groups. The phylum Fusobacteria was more prevalent in fibrotic ILD than in nonfibrotic ILD. Inter-sample differences in relative abundances were more prominent in BALF versus sputum specimens. *Rothia* and *Veillonella* were more prevalent in the sputum than in BALF. We did not detect site-specific dysbiosis in the ILD lung. BALF was an effective respiratory specimen type for evaluating the lung microbiome in patients with ILD. Further studies are needed to evaluate the causal links between the lung microbiome and the pathogenesis of ILD.

## 1. Introduction

Interstitial lung disease (ILD) refers to various chronic, progressive pulmonary disorders involving interstitial proliferation and fibrosis with diverse known or unknown etiologies.^[1]^ Idiopathic pulmonary fibrosis (IPF), the most prevalent type of idiopathic interstitial pneumonia, is associated with progressive lung function decline and poor clinical outcomes.^[2]^ Repetitive alveolar epithelial injury is a key initiating factor in the pathogenesis of ILD.^[3–5]^ If several stimuli (e.g., smoking, gastric acid, and infections) recurrently injure alveolar epithelial cells, the subsequent dysregulated repair process results in interstitial inflammation and irreversible fibrosis in predisposed patients. Research on risk factors for IPF has focused on dust, pollution, asbestosis, and gastric aspirates. Relatively little work has evaluated the contribution of infections; however, the adverse effects of immunosuppressants on IPF outcomes suggest that infection has an essential role.^[6]^

Previous studies have shown that viral infections are associated with the development of IPF.^[7–9]^ In addition, there is evidence that antiviral treatment results in disease stabilization in a small number of patients with IPF who have positive Epstein-Barr virus serology, despite short study periods.^[10]^ However, the causality between viral infection and the development of IPF is unclear. Given the classical notion that the lungs are a sterile organ, few studies have evaluated the role of bacterial infection. However, several studies have suggested that bacterial infections are involved in the progression of IPF. The genera *Haemophilus, Streptococcus, Neisseria*, and *Veillonella* are more prevalent in patients with IPF than in normal subjects.^[11]^ Shulgina et al reported that 12 months of antibiotic treatment reduced the infection rate and mortality in patients with IPF.^[12]^

The development of culture-independent methods (e.g., 16S ribosomal RNA sequencing) has enabled the detection of the lung microbial community,^[13,14]^ termed the microbiome. The human microbiome refers to a set of genomes derived from all microorganisms inhabiting the body surface or organs.^[15]^ Recent studies have suggested that there is a relationship between chronic lung diseases and the respiratory microbiome.^[16–18]^ An altered lung microbiome may be associated with the development and progression of IPF. Molyneaux et al observed that an increased intrapulmonary bacterial burden is associated with acute exacerbation of IPF.^[19]^ Frequencies of *Streptococcus* or *Staphylococcus* species above certain thresholds in lower airway specimens from patients with stable IPF are associated with IPF progression.^[20]^

However, a causal relationship between the lung microbiome and the pathogenesis of ILD has not yet been clearly established. The aim of the current study was to evaluate characteristics of respiratory microbiome in patients with ILD as well as to determine intrasubject differences (i.e., differences among the sputum and segmental bronchus where ILD-related parenchymal changes were minor and severe).

## 2. Materials and methods

### 2.1. Study population

Adult (≥19 years) patients with clinically and radiologically diagnosed ILD who visited our institution (800-bed university-affiliated tertiary hospital) between January 2021 and December 2021 were enrolled. Patients were excluded if they received recent antibiotic or systemic steroid treatment (≤3 months). Patients with active cardiac disease (i.e., acute coronary syndrome, uncontrolled arrhythmias, or heart failure), pregnancy, documented advance directives, or terminal illness were also excluded. Enrolled patients were hospitalized and blood sampling, physiologic tests (pulmonary function test and 6-minute walk test), and bronchoscopy were performed. Baseline demographics, including occupational and drug history, pets, hobbies, and environmental exposure, were investigated through a questionnaire survey. Chest high-resolution computed tomography (CT) was conducted to evaluate radiologic patterns of ILD in all participants. Fibrotic ILD was defined when at least 1 of the fibrotic features (bilateral reticulation, traction bronchiectasis, or honeycombing) was noted. Lung tissues via transbronchial lung biopsy or surgical lung biopsy were obtained if needed for a definitive diagnosis.

### 2.2. Collection of respiratory specimens

Sputum was collected after mouth rinsing with sterile saline. Sputum induction with 5 mL of nebulized 3% saline was performed if patients had difficulty with expectoration. On the day of and the day before bronchoscopy, the patients gargled twice with 15 mL of chlorhexidine solution. Experienced pulmonologists performed bronchoscopy procedures under conscious sedation. The following bronchoscopes (Olympus, Tokyo, Japan) with outer diameters of 5.9 or 4.9 mm were used: BF-1T260 and BF-260.

Bronchoalveolar lavage (BAL) was performed twice with instillations of 50 mL of sterile saline in 2 different sites. First, BAL was performed in the segmental bronchus where ILD-related parenchymal changes were most severe; BAL fluid (BALF) was labeled marked BALF-1. Suctions through the bronchoscope channel were not permitted before the first BAL. A second BAL was performed in the segmental bronchus where ILD-related parenchymal changes were least severe; BALF was labeled BALF-2. The second BAL procedure was performed using another bronchoscope. At least 15 mL of BALF was obtained from each site, and at least 5 mL of BALF was used for the metagenomics analysis. Collected samples (sputum, BALF-1, and BALF-2) were immediately stored in a refrigerator at −70°C.

### 2.3. Sample preparation, library construction, and sequencing

DNA was extracted from each sample within 24 hours after the specimen acquisition. DNA extraction protocols are detailed in the Supplemental Digital Content 1, http://links.lww.com/MD/I739. After quality control, qualified samples (concentration of extracted DNA > 10 ng/μL) were used for library construction.

The sequencing libraries are prepared according to the Illumina 16S Metagenomic Sequencing Library protocols to amplify the V3 and V4 region. The input gDNA 2ng was Polymerase chain reaction (PCR) amplified with 5ⅹ reaction buffer, 1 mM of dNTP mix, 500 nM each of the universal F/R PCR primer, and Herculase II fusion DNA polymerase (Agilent Technologies, Santa Clara, CA). The cycle condition for 1st PCR was 3 minutes at 95°C for heat activation, and 25 cycles of 30 seconds at 95°C, 30 seconds at 55°C and 30 seconds at 72°C, followed by a 5-minutes final extension at 72°C. The universal primer pair with Illumina adapter overhang sequences used for the first amplifications were as follows:

16S Amplicon PCR Forward Primer 5′ (TCGTCGGCA GCGTCAGATG TGTATAAGAGA CAGCCTAC GGGNGGC WGCAG),

16S Amplicon PCR Reverse Primer 5′ (GTCTCGTGGGC TCGGAGATGT GTATAAGAGACA GGACTACHVG GGTATCTAATCC).

The 1st PCR product was purified with AMPure beads (Agencourt Bioscience, Beverly, MA). Following purification, the 2 μL of 1st PCR product was PCR amplified for final library construction containing the index using NexteraXT Indexed Primer. The cycle condition for 2nd PCR was same as the 1st PCR condition except for 10 cycles. The PCR product was purified with AMPure beads. The final purified product is then quantified using qPCR according to the qPCR Quantification Protocol Guide (KAPA Library Quantificatoin kits for Illumina Sequecing platforms) and qualified using the TapeStation D1000 ScreenTape (Agilent Technologies, Waldbronn, Germany). And then we sequenced using the MiSeq platform (Illumina, San Diego).

### 2.4. Amplicon sequence variants (ASVs) analysis

After sequencing is completed, we classified Illumina MiSeq raw data using the index sequence. In order to correct errors in the amplicon sequencing process, DADA2 program was used.^[21]^ After assembling the error-corrected paired-end sequences into 1 sequence, Chimera sequences were removed and ASVs were formed by using the Consensus method of DADA2. Each ASV sequences were assigned to the taxonomic information for organism with highest similarity based on reference data base (NCBI 16s microbial data base). Comparative analysis of various microbial communities was performed by using QIIME^[22]^ with ASVs abundance and taxonomy information.

We used a Rarefaction curve graph to evaluate whether a sufficient amount of sequencing was done for data analysis. Alpha rarefaction graph shows whether the number of reads used in analysis was sufficient in identifying species/operational taxonomic unit (OTU). If the curve becomes flatter to the right, it indicates that a reasonable number of reads have been used in the analysis, thus additional sequencing is not necessary. In contrast, if the graph does not plateau, the additional reads is likely to discover more OTUs for the sample (x-axis: read number; y-axis: number of OTUs). Below each plot is a table displaying average values for each measure of alpha diversity for each group of samples the specified category. A series of procedures were performed at Macrogen, Inc. (Seoul, Korea).

### 2.5. Statistical analysis

Shannon, Gini-Simpson, ASVs, and Chao1 diversity indexes were used to evaluate species richness in the microbiome of a respiratory sample (α-diversity). Unweighted and weighted UniFrac distances were calculated to evaluate differences between samples in different groups (β-diversity). Groups were compared using the Wilcoxon rank sum test.^[23]^ R software (version 3.6.2) was used for statistical analyses. A *P* value of <.05 was considered statistically significant.

### 2.6. Study ethics

This study was approved by the Institutional Review Board and Ethics Committee of our institution (institutional review board number: CBNUH 2020-11-001-001) and was conducted in compliance with the Declaration of Helsinki. Written informed consent was obtained from all patients.

## 3. Results

### 3.1. Patient recruitment and characteristics

**Figure 1.**
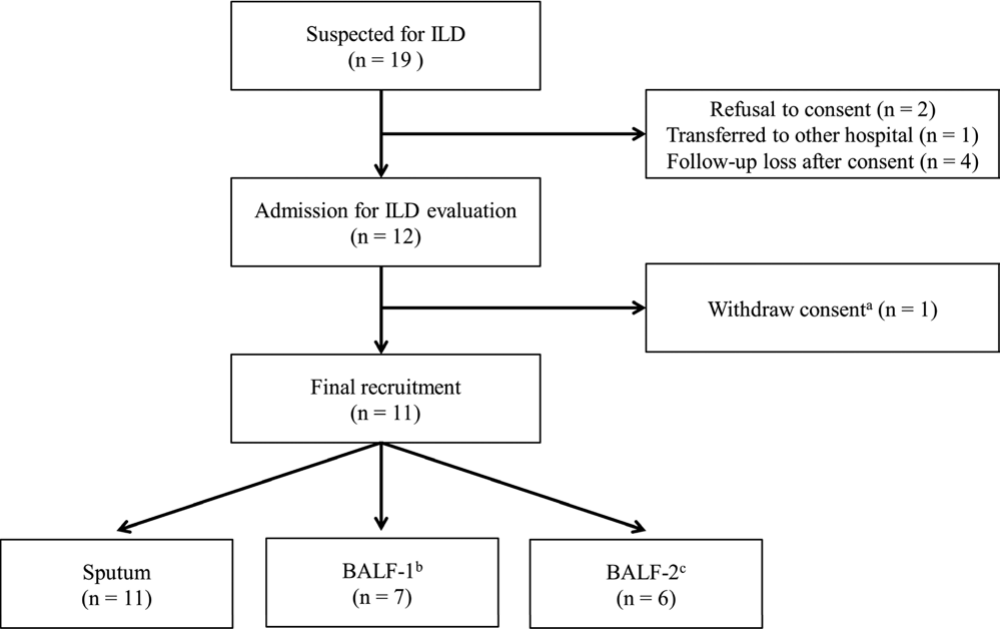
Flowchart of patient recruitment. BALF = bronchoalveolar lavage fluid, ILD = interstitial lung disease. ^a^The patient was withdrawn from the study owing to acute exacerbation of ILD occurring immediately after hospital admission. ^b^BALF obtained from the related segmental bronchus in which ILD-related parenchymal changes were most severe. ^c^BALF obtained from the related segmental bronchus in which ILD-related parenchymal changes were least severe.

We screened 19 patients with suspected ILD and ultimately enrolled 11 patients in the study (Fig. 1). Thirty-three respiratory samples were obtained, that is, 3 samples (sputum, BALF-1, and BALF-2) from 11 patients. Finally, 24 samples passed quality control and were subjected to sequencing: sputum (n = 9), BALF-1 (n = 7), BALF-2 (n = 6).

The mean age was 70.1 ± 7.9 years; 36.4% were women and 90.9% were current or former smokers. The mean forced vital capacity and diffusing capacity of the lungs for carbon monoxide were 75.3% and 59.2% of predicted, respectively. Trans-bronchial lung biopsy (n = 8, 72.7%) and surgical lung biopsy (n = 2, 18.2%) were performed. Final diagnoses were as follows: organizing pneumonia (OP) (n = 4, 36.4%), IPF (n = 3, 27.3%), connective tissue disease related-ILD (CTD-ILD) (n = 2, 18.2%), and nonspecific interstitial pneumonia (NSIP) (n = 2, 18.2%). Patient demographics are shown in Table 1.

**Table 1:** Baseline characteristics of enrolled patients.

Variables	Total (n = 11)
Female sex, n (%)	4 (36.4)
Age, yr	70.1 (7.9)
Body mass index, kg/m^2^	24.8 (2.9)
Current or former smoker	10 (90.9)
Gastroesophageal reflux disease	2 (18.2)
Dyspnea scale by the modified Medical Research Council grade	
0 or 1	8 (72.7)
≥2	3 (27.3)
Physiologic parameters	
Six-min walk distance*, m	408.9 (130.5)
Forced vital capacity, % predicted	75.3 (17.4)
Diffusion capacity of lung for carbon monoxide†, % predicted	59.2 (17.2)
Radiologic patterns on computed tomography	
Ground-glass opacity	11 (100)
Consolidation	3 (27.3)
Reticulation	6 (54.4)
Traction bronchiectasis	5 (45.5)
Honeycombing	3 (27.3)
Lymphocytosis of bronchoalveolar lavage fluid, %	22.6 (21.7)
Trans-bronchial lung biopsy	2 (18.2)
Surgical lung biopsy	2 (18.2)
Final diagnosis	
Organizing pneumonia‡	4 (36.4)
Idiopathic pulmonary fibrosis	3 (27.3)
Connective tissue disease related§	2 (18.2)
Nonspecific interstitial pneumonia∥	2 (18.2)

Data are presented as n (%) or mean (standard deviation).

* Nine patients were tested for each parameter.

† Nine patients were tested for each parameter.

‡ Cryptogenic (n = 3), secondary due to coronavirus-19 (n = 1).

§ Systemic sclerosis (n = 1), Sjogren syndrome (n = 1).

∥ Idiopathic or unknown cause.

### 3.2. Intrasubject differences in the lower airway microbiome

**Figure 2.**
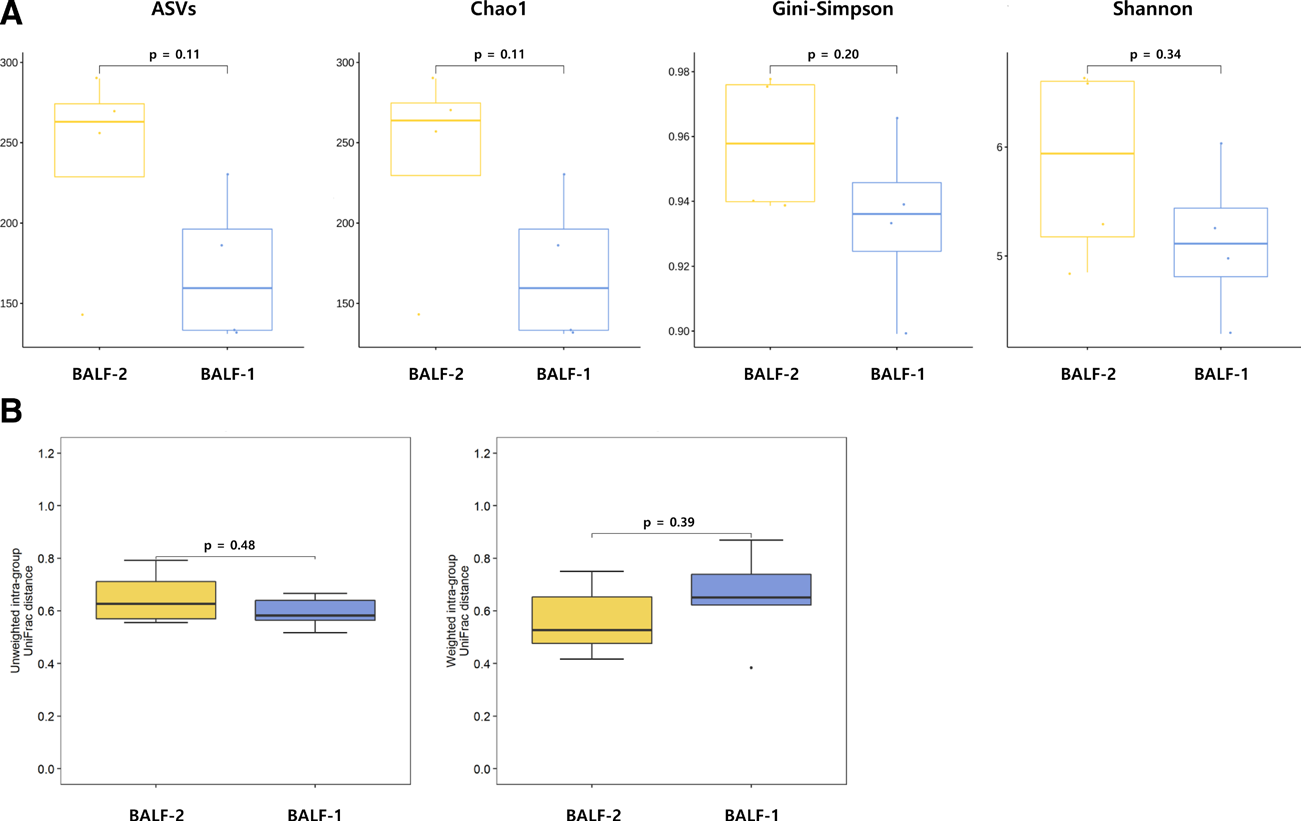
Intrasubject comparisons of diversity indices for the lower airway microbiome. α-diversity (A) and β-diversity (B) were similar between BALF-1 and BALF-2. However, α-diversity tended to be lower in BALF-1 than in BALF-2. BALF = bronchoalveolar lavage fluid.

**Figure 3.**
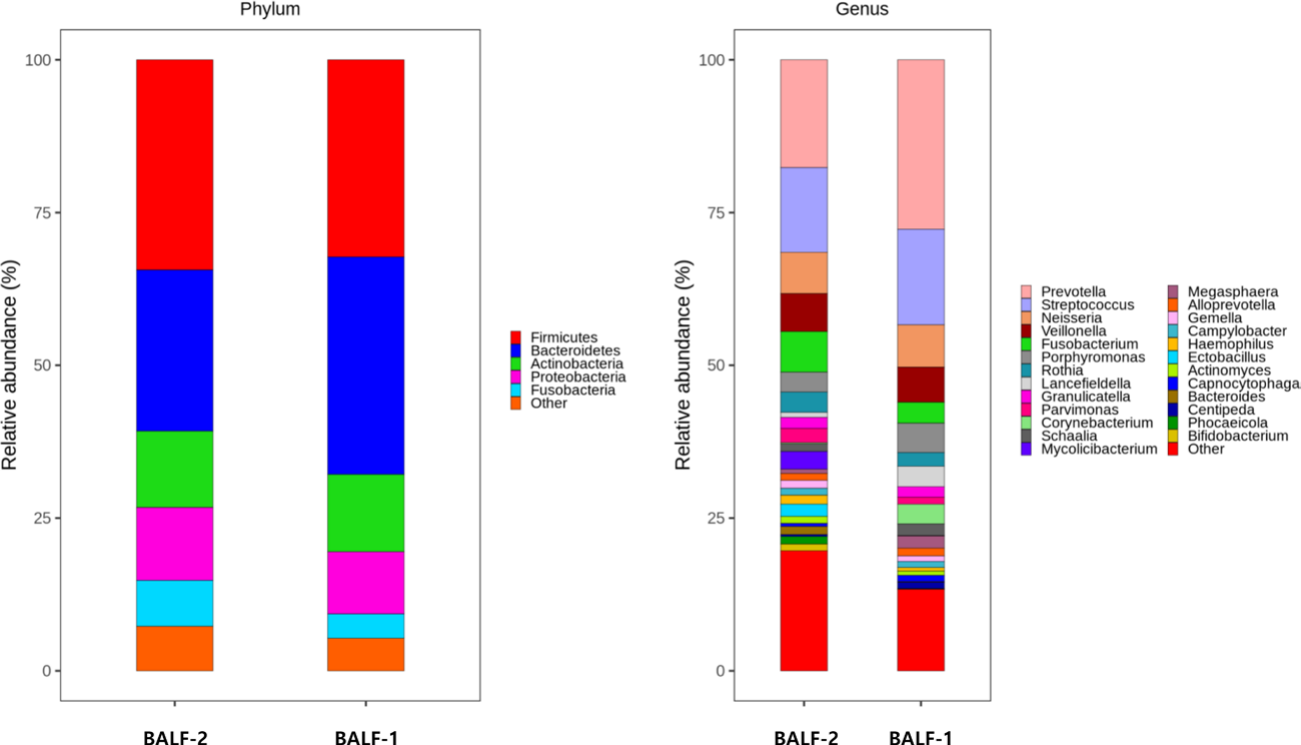
Relative abundances of taxa in the lower airway microbiome in BALF-1 and BALF-2 at the phylum and genus level. Nondominant taxa with relative abundance estimates of <1% are indicated as other. BALF = bronchoalveolar lavage fluid.

Taxonomic diversity was compared between BALF-1 and BALF-2 samples (Fig. 2). Paired samples obtained from 4 patients were used: IPF (n = 2), NSIP (n = 1), OP (n = 1). In these patients, BALF-1 and BALF-2 were obtained from the left lower lobe bronchus and the left upper lobe bronchus, respectively. Although the difference was not statistically significant, α-diversity (indicators of mean species diversity) tended to be higher in BALF-2 (the least affected lesion) than in BALF-1 (the most affected lesion). There was no significant difference between BALF-1 and BALF-2 in β-diversity (the extent of change in communities or degree of community differentiation). Taxonomic abundance results at the phylum and genus levels in BALF-1 and BALF-2 are shown in Figure 3. At the phylum level, Firmicutes (34.4%) was the most common, followed by Bacteroidetes (26.4%) and Actinobacteria (12.5%) in BALF-2. Bacteroidetes (35.6%) was the most common, followed by Firmicutes (32.3%) and Actinobacteria (12.7%) in the BALF-1 group. At the genus level, taxonomic abundances were similar in the 2 groups. *Prevotella* (BALF-1: 27.7%, BALF-2: 17.7%) was the most common, followed by *Streptococcus* (BALF-1: 15.7%, BALF-2: 13.8%) and *Neisseria* (BALF-1: 6.9%, BALF-2: 6.8%). However, no significant difference in relative abundance was observed between the 2 groups. Taxonomic abundance data are detailed in Table S1, Supplemental Digital Content 2, http://links.lww.com/MD/I740.

### 3.3. Differences in the lower airway microbiome between nonfibrotic and fibrotic ILD

**Figure 4.**
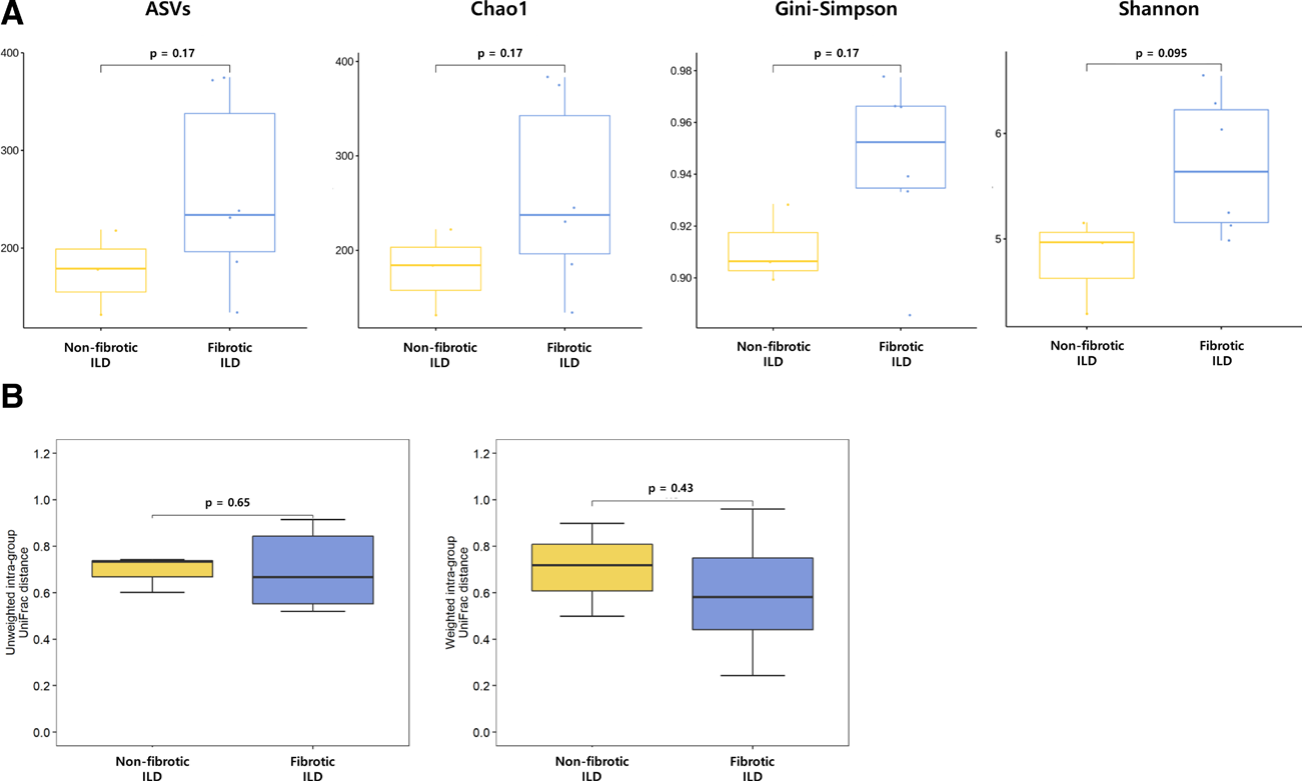
Comparisons of diversity indices in the lower airway microbiome between nonfibrotic and fibrotic ILD. α-diversity (A) and β-diversity (B) were similar in the 2 groups. However, α-diversity tended to be lower in nonfibrotic ILD than in fibrotic ILD. ILD = interstitial lung disease.

**Figure 5.**
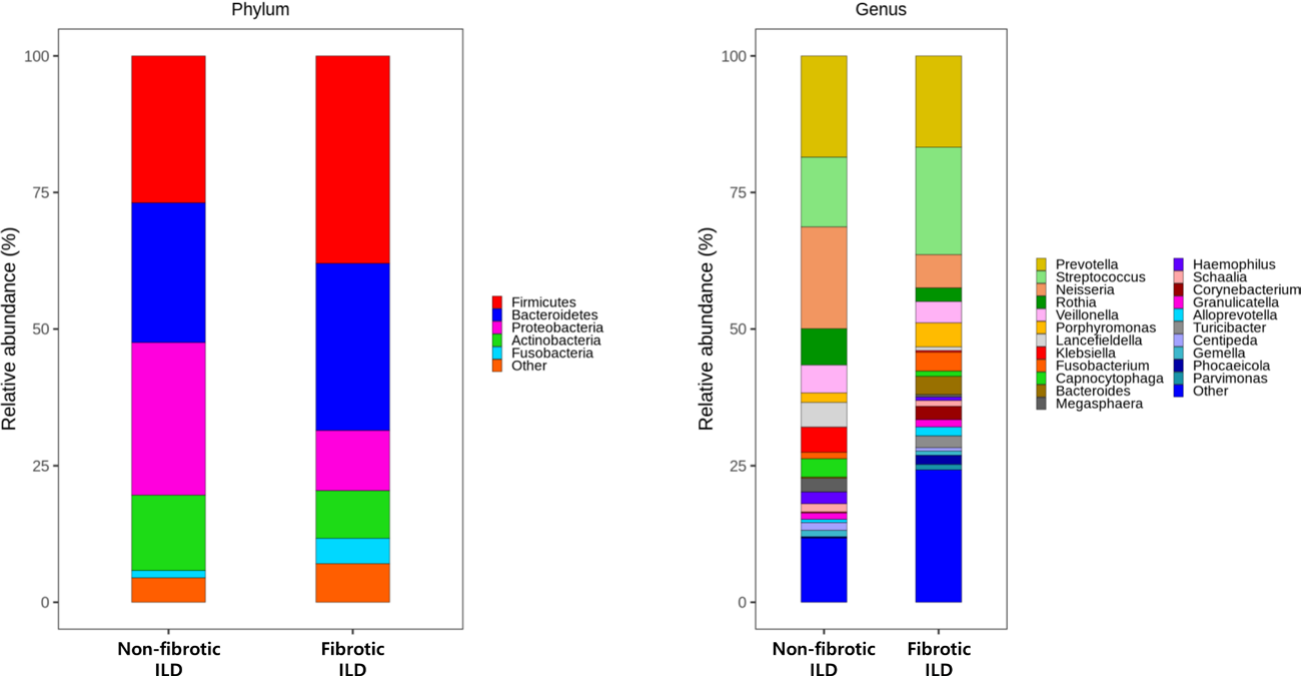
Relative abundance estimates for taxa in the lower airway microbiome in nonfibrotic and fibrotic ILD at the phylum and genus levels. nondominant taxa with relative abundance estimates of < 1% are indicated as other. ILD = interstitial lung disease.

BALF samples obtained from nonfibrotic [OP (n = 3)] and fibrotic ILD [CTD-ILD (n = 1), IPF (n = 3), NSIP (n = 2)] were used for analyses. The α-diversity indexes tended to be higher in fibrotic ILD than in nonfibrotic ILD; however, the differences were not significant. ß-diversity was similar in the 2 groups (Fig. 4). Relative abundance at the phylum and genus levels between 2 groups is shown in Figure 5. At the phylum level, Proteobacteria (27.9%) was the most abundant in nonfibrotic ILD, whereas Firmicutes (38.0%) was the most abundant in fibrotic ILD. Fusobacteria was significantly more prevalent in the nonfibrotic ILD group than in the fibrotic ILD group (4.6% vs 1.3%, *P* = .048) (Figure S1, Supplemental Digital Content 3, http://links.lww.com/MD/I741). At the genus level, *Neisseria* (18.6%) and *Prevotella* (18.5%) were dominant in nonfibrotic ILD, whereas *Streptococcus* (19.7%) was the most prevalent taxon in fibrotic ILD. Taxonomic abundance did not differ significantly between the 2 groups, except for several minor taxa (<1%). Taxonomic abundance data are detailed in Table S2, Supplemental Digital Content 4, http://links.lww.com/MD/I742.

### 3.4. Differences in the microbiome community between the sputum and BALF

Paired samples obtained from 9 patients were used: IPF (n = 3), OP (n = 3), NSIP (n = 2), and CTD-ILD (n = 1). Estimates of α-diversity were similar between sputum and BALF specimens. However, ß-diversity was significantly higher in the BALF than in the sputum (Figure S2, Supplemental Digital Content 5, http://links.lww.com/MD/I743). Relative abundances at the phylum and genus levels in the 2 groups are summarized in Figure S3, Supplemental Digital Content 6, http://links.lww.com/MD/I744. At the phylum level, Firmicutes was the most prevalent taxa in both groups, that is, sputum (47.2%) and BALF (34.3%). Bacteroidetes was significantly more abundant in BALF than in sputum (28.9% vs 13.1%, *P* = .011), whereas Firmicutes was more abundant in sputum than in BALF (47.2% vs 34.3%, *P* = .019) (Figure S4, Supplemental Digital Content 7, http://links.lww.com/MD/I745). At the genus level, *Streptococcus* was the most prevalent taxon in both groups, i.e., sputum (24.6%) and BALF (17.4%). *Rothia* (12.8% vs 3.9% *P* = .04) and *Veillonella* (15.6% vs 4.3%, *P* = .014) were significantly more abundant in sputum than in BALF. *Bacteroides* was significantly more abundant in BALF than in sputum (0.01% vs 2.3%, *P* = .003). Taxonomic abundance data are detailed in Table S3, Supplemental Digital Content 8, http://links.lww.com/MD/I746.

## 4. Discussion

The aim of the current study was to characterize the respiratory microbiome and intrasubject variation in patients with ILD. The sample size was insufficient owing in part to the COVID-19 pandemic during the study period. Moreover, the burdensome study protocol (i.e., BAL procedures in the setting of ILD) may result in delayed recruitment because BAL is not mandatory for the diagnosis of ILD.^[24]^ However, to the best of our knowledge, this is the first study of intrasubject differences of the lower airway microbiome in patients with ILD.

We did not detect consistent intrasubjective differences in the lower airway microbiome. The microbial composition was similar, regardless of the severity of ILD-related parenchymal changes. In a study of healthy subjects, BALF obtained from multiple distal airways did not show distinct microbiome communities.^[25]^ However, mean species richness tended to be lower in lung lesions with prominent ILD-related changes. These results do not indicate the presence of site-specific dysbiosis within an ILD lung. Dysbiosis (i.e., an imbalance between beneficial and harmful microbes in a specified region) encompasses 3 main features: a loss of beneficial microbes, expansion of pathogens, and loss of diversity.^[26]^ Dysbiosis-induced chronic inflammation, metabolic effects, and genotoxicity are potential mechanisms underlying chronic lung disease.^[27]^

Our data did not demonstrate the causality between the lung microbiome and pathogenesis of ILD. However, the reduced species richness in more advanced fibrotic lung lesions may have several explanations. Since all lesions identified as “most affected” were lower lobe lesions (common dominant lung lesions in fibrotic ILDs), microbial migration from an oropharyngeal cavity or gastric acid aspiration would easily affect the results. Moreover, fibrotic lung lesions where ventilation is relatively poor may have a microbial community with low diversity. This is consistent with the fact that the abundance of the genus *Prevotella* was higher in more affected, fibrotic lung lesions than in less affected lesions. *Prevotella*, a genus of anaerobic gram-negative bacteria, is one of the most abundant genera in the human oral cavity with a key player in host-microbiome interactions.^[28]^

Nonfibrotic ILD tended to show lower species diversity than that in fibrotic ILD. All diagnosed nonfibrotic ILD cases were OP in which infection is a major cause.^[3]^ Among 3 patients with OP, 1 case was due to COVID-19 and 2 cases were clinically considered cryptogenic without surgical lung biopsy. Thus, bacterial infection might contribute to the pathogenesis of OP in these patients. Fusobacteria was more prevalent in fibrotic ILD than in nonfibrotic ILD. This phylum included commensal anaerobic gram-negative rods inhabiting the upper respiratory and gastrointestinal tracts, occasionally cause human infectious diseases.^[29]^ Previous studies showed an association between *Fusobacterium* abundance and ILD.^[30,31]^ The role of Fusobacteria in the pathogenesis of ILD should be evaluated further.

Both sputum and BALF showed similar microbial compositions and diversity. However, the phylum Firmicutes and genera *Rothia* and *Veillonella* were significantly more prevalent in the sputum than in BALF. These bacterial taxa are commonly observed in oropharyngeal samples from patients with chronic lung disease.^[32–34]^ Oropharynx-derived microbes are the main components of the lung microbial community.^[25]^ Moreover, ILD-affected lung lesions may have a relatively low biomass. Given that intrasample differences were dominant in BALF (i.e., β-diversity), BALF better reflects the characteristics of the respiratory microbiome than does sputum.

Current data do not support the causal link between the lung microbiome and the pathogenesis and progression of ILD. Therapeutic options (e.g., antifibrotic agents and lung transplantation) are limited for progressive fibrotic ILD, including IPF. The detailed alterations in the lung microbiome may provide a basis for the development of treatment strategies. For example, the identification of subgroups of patients with fibrotic ILD in whom dysbiosis-targeted treatment (e.g., antimicrobial agents) is expected to inhibit disease progression has the potential to substantially improve prognosis. Future large-scale studies that evaluate longitudinal changes in the lung microbiome and measure primary intrapulmonary markers for the pathogenesis of ILD are therefore needed.

Our study had several limitations. First, owing to the lack of comparison arms (healthy subjects or patients with chronic lung diseases other than ILD) and the small sample size, generalization is difficult. Second, oropharyngeal microbe contamination through the bronchoscope channel could not be excluded. However, pharyngeal contamination does not affect bronchoscopy-derived specimens substantially.^[35]^ To address this issue, we used a new, disinfected bronchoscope before the subsequent BAL. A recent study has reported that qualified and acceptable BALF could be obtained from patients with ILD using a balloon catheter.^[36]^ It is necessary to establish a standardized BAL protocol for lung microbiome studies. Third, several samples (mainly in BALF) failed to pass quality control for subsequent library construction. This can be explained by the low biomass of fibrotic lung lesions or DNA degradation during sample transport.

## 5. Conclusions

This study demonstrated that the lower airway microbial community may be affected by the oropharyngeal microbiome in patients with ILD. However, intrasubjective differences were not prominent as expected. Altered respiratory microbiome has been attracting attention as an important role in the pathogenesis of chronic lung diseases such as ILD. Future large-scale studies are needed to elucidate site-specific dysbiosis in an ILD-involved lung. These approaches would be crucial to prove the causality between an altered lung microbiome and ILD.

This work was supported by the National Research Foundation of Korea (NRF) grant funded by the Korea government (MISP) (2020R1A5A2017476). Authors did not have any writing assistance.

## Author contributions

**Conceptualization:** Jun Yeun Cho, Ji Hyoun Kim, Hyeran Kang, Ki Man Lee, Kang Hyeon Choe, Yoon Mi Shin.

**Data curation:** Jun Yeun Cho, Mi Yeon Kim, Ji Hyoun Kim, Eung-Gook Kim, Sun-Hyung Kim, Ki Man Lee, Kang Hyeon Choe, Yoon Mi Shin.

**Formal analysis:** Jun Yeun Cho, Bumhee Yang, Hyeran Kang, Yoon Mi Shin.

**Funding acquisition:** Jun Yeun Cho, Eung-Gook Kim, Yoon Mi Shin.

**Investigation:** Jun Yeun Cho, Mi Yeon Kim, Ji Hyoun Kim, Eung-Gook Kim, Sun-Hyung Kim, Bumhee Yang, Hyeran Kang, Ki Man Lee, Yoon Mi Shin.

**Methodology:** Jun Yeun Cho, Mi Yeon Kim, Bumhee Yang, Hyeran Kang, Kang Hyeon Choe, Yoon Mi Shin.

**Project administration:** Jun Yeun Cho.

**Resources:** Jun Yeun Cho, Ji Hyoun Kim, Eung-Gook Kim, Sun-Hyung Kim, Bumhee Yang, Ki Man Lee, Kang Hyeon Choe, Yoon Mi Shin.

**Software:** Hyeran Kang.

**Supervision:** Ji Hyoun Kim, Eung-Gook Kim, Sun-Hyung Kim, Bumhee Yang, Ki Man Lee, Kang Hyeon Choe, Yoon Mi Shin.

**Validation:** Jun Yeun Cho, Mi Yeon Kim, Eung-Gook Kim, Sun-Hyung Kim, Bumhee Yang, Hyeran Kang, Ki Man Lee, Kang Hyeon Choe, Yoon Mi Shin.

**Visualization:** Jun Yeun Cho, Mi Yeon Kim, Yoon Mi Shin.

**Writing – original draft:** Jun Yeun Cho, Yoon Mi Shin.

**Writing – review & editing:** Jun Yeun Cho, Ji Hyoun Kim, Eung-Gook Kim, Sun-Hyung Kim, Bumhee Yang, Hyeran Kang, Ki Man Lee, Kang Hyeon Choe, Yoon Mi Shin.

## Supplementary Material
