# Supplementary material for: Characteristics and intrasubject variation in the respiratory microbiome in interstitial lung disease
Source: Medicine (Baltimore). 2022 Apr 7;102(14):e33402. doi: 10.1097/MD.0000000000033402 (PMC10082288; doi:10.1097/MD.0000000000033402)
Supplement: Supplementary file 1 [file medi-102-e33402-s001.pdf]

Soil DNA Extraction protocol

1. Add soil sample to the PowerBead Tube provided. (solid : 0.25g, liquid : 300μl)
2. Add 60μl of Solution C1. (Solution C1 may be added to the PowerBead tube before adding soil sample)
3. Secure PowerBead Tubes horizontally using a Vortex Adapter tube holder and vortex at maximum speed for 10 min.
4. Centrifuge tubes at 10,000xg for 1 min.
5. Transfer the supernatant to a clean 2 ml collection tube.
6. Add 250μl of Solution C2 and vortex for 5 s. Incubate at 4°C for 5 min. (Before transfer supernatant, add solution C2 to the clean tube.)
7. Centrifuge tubes at 10,000xg for 1 min.
8. Avoiding the pellet, transfer up to 600μl of supernatant to a clean 2ml collection tube.
9. Add 200μl of Solution C3 and vortex briefly. Incubate at 2–8°C for 5 min. (Before transfer supernatant, add solution C3 to the clean tube.)
10. Centrifuge tubes at 10,000xg for 1 min.
11. Avoiding the pellet, transfer up to 750μl of supernatant to a clean 2ml collection tube.
12. Add 1200μl of solution C4 and pipetting. (Before transfer supernatant, add solution C4 to the clean tube.)

    -Centrifuge-

13. Load 675μl onto an MB Spin Column and centrifuge at 10,000xg for 1 min. Discard flow through.
14. Repeat step 13, until all of the sample has been processed.
15. Add 500μl of Solution C5. Centrifuge for 30 s at 10,000xg.

-Vacuum-

13. Place MB spin column on vacuum.
14. Put samples in column and operate pump.
15. Add 500μl of Solution C5 and operate pump.
16. Transfer column to empty collection tube and centrifuge at Max speed for 1 min. (to dry)
17. Transfer column to 1.5ml tube and add 30-100μl Solution C6.
18. Centrifuge at Max speed 1 min for elution.
