# Supplementary material for: Characteristics and intrasubject variation in the respiratory microbiome in interstitial lung disease
Source: Medicine (Baltimore). 2022 Apr 7;102(14):e33402. doi: 10.1097/MD.0000000000033402 (PMC10082288; doi:10.1097/MD.0000000000033402)
Supplement: Supplementary file 2 [file medi-102-e33402-s002.pdf]

Supplemental table 1. Taxonomic abundance between BALF-1 and BALF-2

| **Phylum** | **BALF-2** | **BALF-1** |
|---|---|---|
| Firmicutes | 34.39 | 32.27 |
| Bacteroidetes | 26.37 | 35.58 |
| Actinobacteria | 12.50 | 12.67 |
| Proteobacteria | 11.93 | 10.17 |
| Fusobacteria | 7.51 | 3.98 |
| Other | 7.30 | 5.34 |
| **Genus** | **Least** | **Most** |
| Prevotella | 17.65 | 27.72 |
| Streptococcus | 13.84 | 15.66 |
| Neisseria | 6.76 | 6.94 |
| Veillonella | 6.22 | 5.72 |
| Fusobacterium | 6.65 | 3.43 |
| Porphyromonas | 3.23 | 4.81 |
| Rothia | 3.33 | 2.24 |
| Lancefieldella | 0.83 | 3.35 |
| Granulicatella | 1.81 | 1.72 |
| Parvimonas | 2.31 | 1.14 |
| Corynebacterium | 0.21 | 3.21 |
| Schaalia | 1.24 | 1.94 |
| Mycolicibacterium | 2.91 | 0.06 |
| Megasphaera | 0.70 | 1.99 |
| Alloprevotella | 1.13 | 1.27 |
| Gemella | 1.29 | 0.87 |
| Campylobacter | 1.14 | 1.00 |
| Haemophilus | 1.45 | 0.60 |
| Ectobacillus | 2.01 | 0.00 |
| Actinomyces | 1.14 | 0.68 |
| Capnocytophaga | 0.50 | 1.02 |
| Bacteroides | 1.34 | 0.11 |
| Centipeda | 0.31 | 1.04 |
| Phocaeicola | 1.24 | 0.11 |
| Bifidobacterium | 1.11 | 0.01 |
| Other | 19.63 | 13.37 |

Data are presented as %
