# Supplementary material for: Characteristics and intrasubject variation in the respiratory microbiome in interstitial lung disease
Source: Medicine (Baltimore). 2022 Apr 7;102(14):e33402. doi: 10.1097/MD.0000000000033402 (PMC10082288; doi:10.1097/MD.0000000000033402)

Supplemental Figure 1. Dominant taxa at phylum level between the fibrotic ILD and non-fibrotic ILD

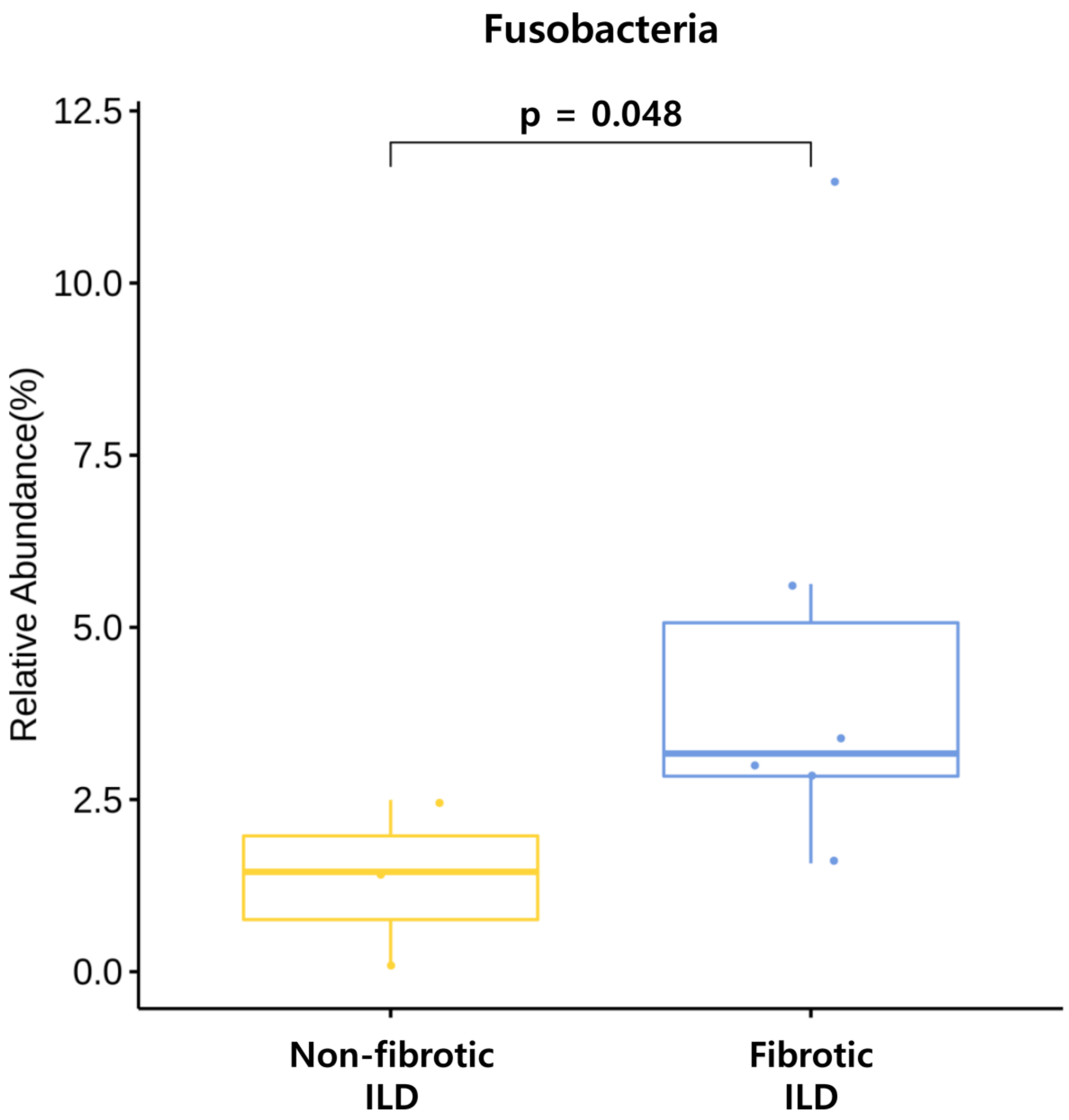

Supplement: Supplementary file 3 [file medi-102-e33402-s003.pdf]
