# Supplementary material for: Characteristics and intrasubject variation in the respiratory microbiome in interstitial lung disease
Source: Medicine (Baltimore). 2022 Apr 7;102(14):e33402. doi: 10.1097/MD.0000000000033402 (PMC10082288; doi:10.1097/MD.0000000000033402)
Supplement: Supplementary file 4 [file medi-102-e33402-s004.pdf]

Supplemental table 2. Taxonomic abundance of lower airway microbiome between fibrotic and non-fibrotic ILD

**Phylum**	**Non-fibrotic_ILD**	**Fibrotic_ILD**
Firmicutes	26.90	37.99
Bacteroidetes	25.55	30.55
Proteobacteria	27.93	11.01
Actinobacteria	13.80	8.72
Fusobacteria	1.33	4.63
Other	4.46	7.06
**Genus**	**Non-fibrotic_ILD**	**Fibrotic_ILD**
Streptococcus	12.77	19.66
Prevotella	18.54	16.72
Neisseria	18.61	6.10
Porphyromonas	1.74	4.36
Veillonella	5.09	3.91
Fusobacterium	1.19	3.46
Bacteroides	0.20	3.31
Rothia	6.63	2.48
Corynebacterium	0.17	2.41
Turicibacter	0.01	2.15
Phocaeicola	0.16	1.64
Alloprevotella	0.56	1.64
Granulicatella	1.18	1.35
Schaalia	1.51	1.07
Parvimonas	0.08	1.00
Capnocytophaga	3.36	0.97
Gemella	1.16	0.81
Lancefieldella	4.52	0.68
Haemophilus	2.14	0.63
Centipeda	1.43	0.59
Megasphaera	2.50	0.46
Klebsiella	4.59	0.27
Other	11.75	24.23

Data are presented as %
