# Supplementary figures and images for: Characteristics and intrasubject variation in the respiratory microbiome in interstitial lung disease
Source: Medicine (Baltimore). 2022 Apr 7;102(14):e33402. doi: 10.1097/MD.0000000000033402 (PMC10082288; doi:10.1097/MD.0000000000033402)

Supplemental Figure 2. Comparisons of diversity indices between sputum and BALF.

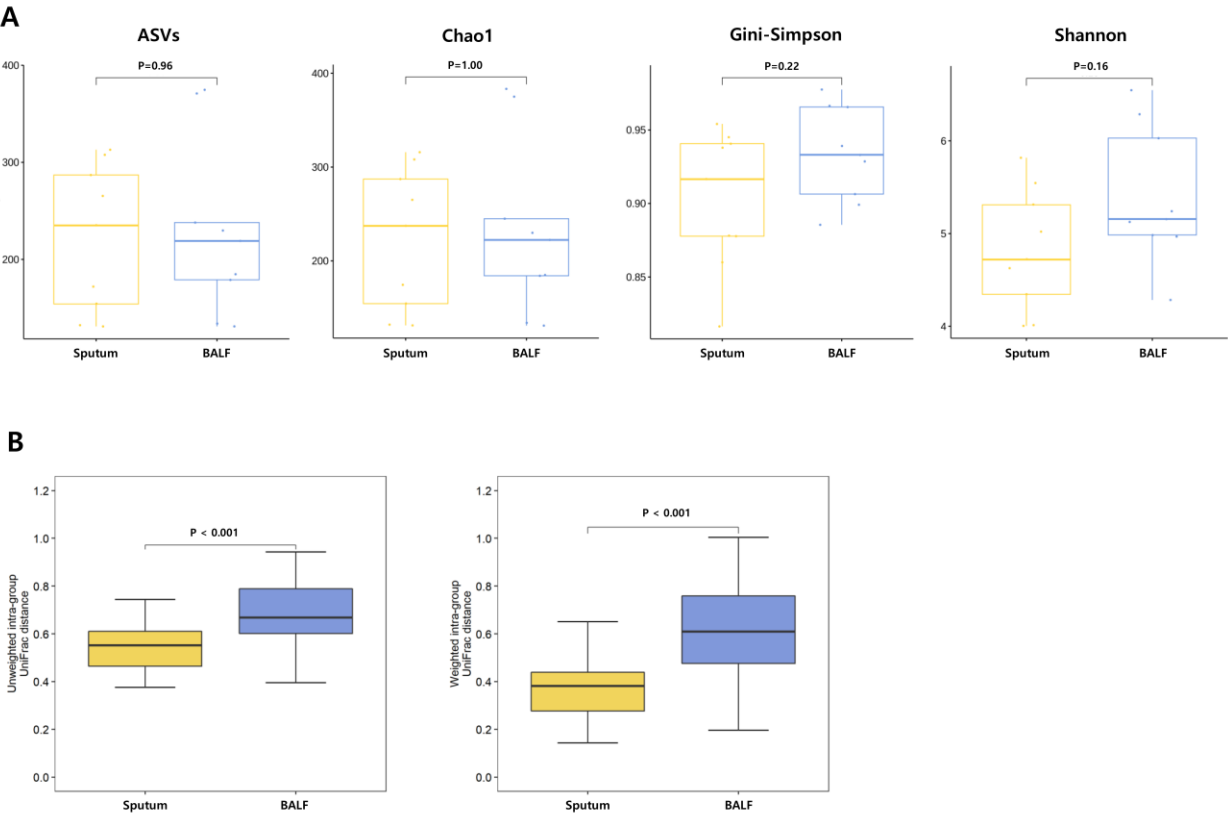

Supplement: Supplementary file 5 [file medi-102-e33402-s005.pdf]

Supplemental Figure 4. Dominant taxa at phylum and genus level between sputum and BALF

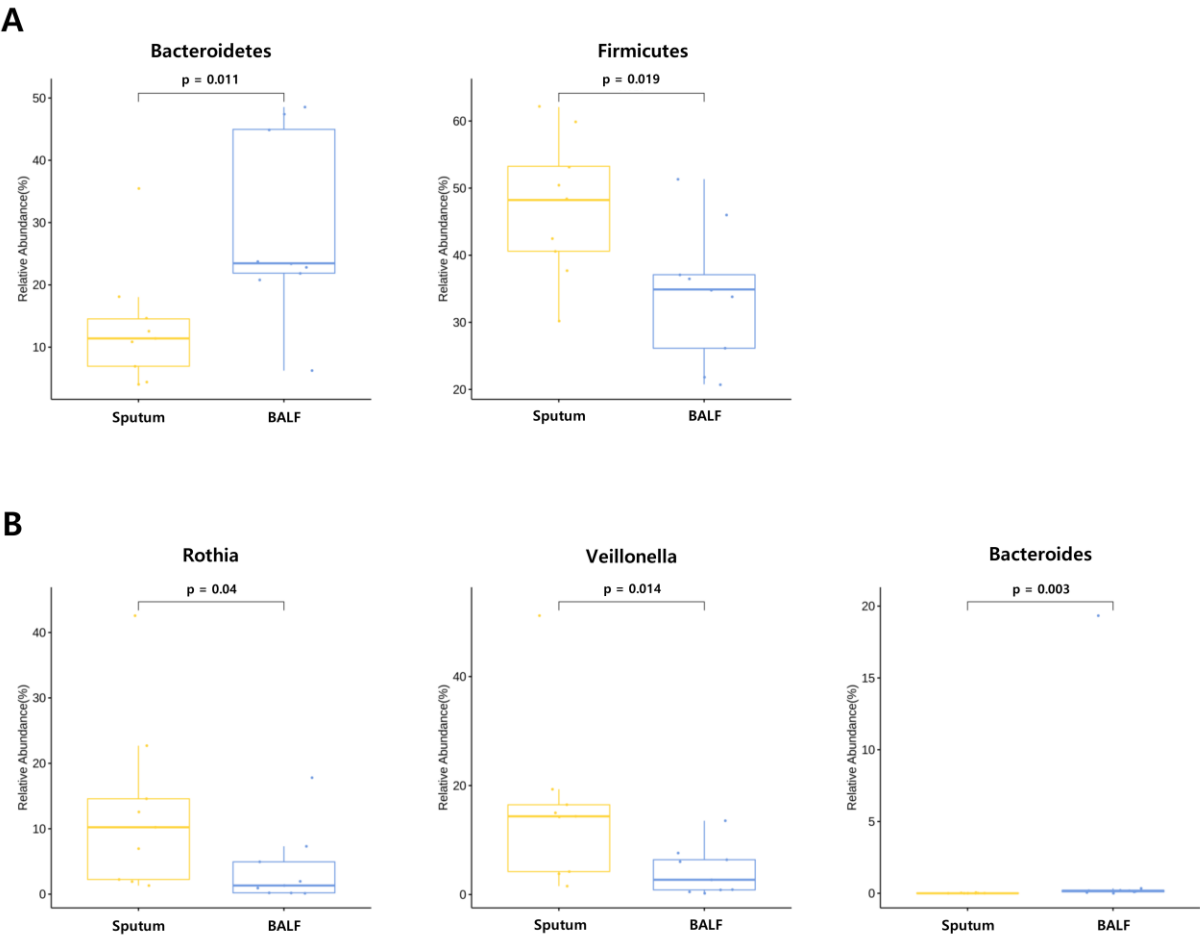

Supplement: Supplementary file 7 [file medi-102-e33402-s007.pdf]
