# Supplementary material for: Characteristics and intrasubject variation in the respiratory microbiome in interstitial lung disease
Source: Medicine (Baltimore). 2022 Apr 7;102(14):e33402. doi: 10.1097/MD.0000000000033402 (PMC10082288; doi:10.1097/MD.0000000000033402)

Supplemental Figure 3. Relative abundance estimates for taxa in sputum and BALF at the phylum and genus levels. Non-dominant taxa with relative abundance estimates of <1% are indicated as other.

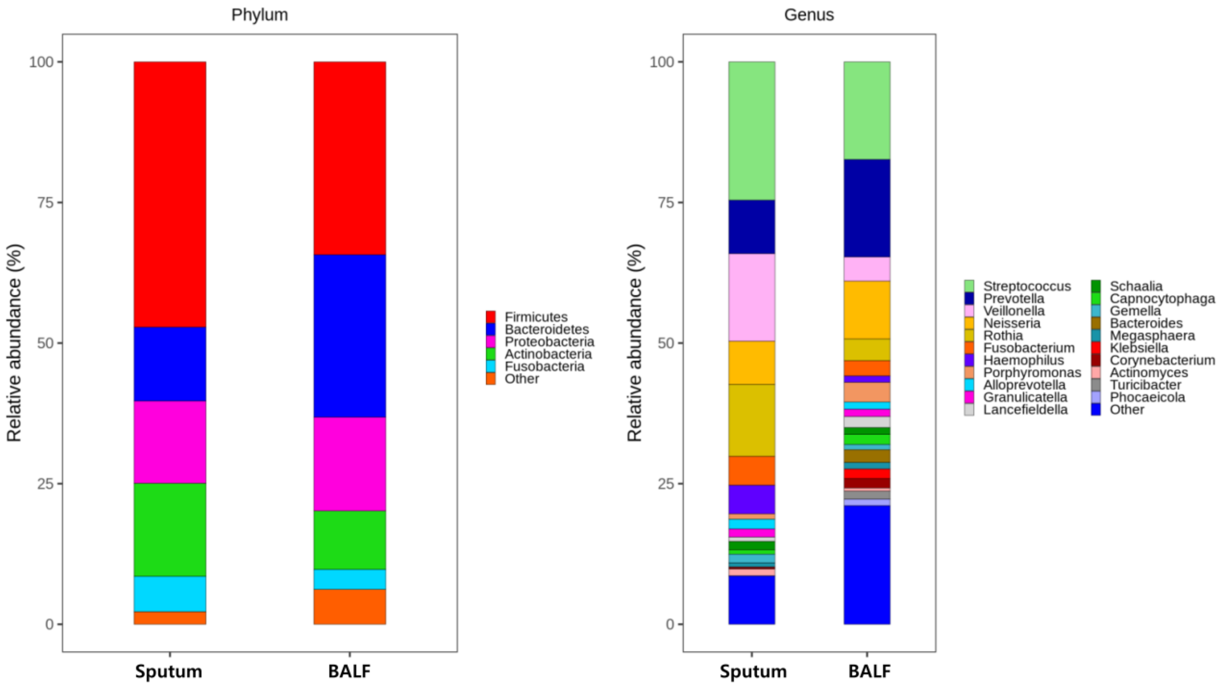

Supplement: Supplementary file 6 [file medi-102-e33402-s006.pdf]
