# Supplementary material for: Characteristics and intrasubject variation in the respiratory microbiome in interstitial lung disease
Source: Medicine (Baltimore). 2022 Apr 7;102(14):e33402. doi: 10.1097/MD.0000000000033402 (PMC10082288; doi:10.1097/MD.0000000000033402)
Supplement: Supplementary file 8 [file medi-102-e33402-s008.pdf]

Supplemental table 3. Taxonomic abundance between sputum and BALF

Phylum	sputum	BALF
Firmicutes	47.18	34.30
Bacteroidetes	13.13	28.88
Proteobacteria	14.62	16.66
Actinobacteria	16.56	10.42
Fusobacteria	6.29	3.54
Other	2.22	6.20
**Genus**	**sputum**	**BALF**
Streptococcus	24.60	17.37
Prevotella	9.51	17.33
Veillonella	15.57	4.30
Neisseria	7.69	10.27
Rothia	12.79	3.87
Fusobacterium	5.13	2.71
Haemophilus	5.08	1.14
Porphyromonas	0.96	3.49
Alloprevotella	1.72	1.29
Granulicatella	1.45	1.30
Lancefieldella	0.78	1.97
Schaalia	1.48	1.22
Capnocytophaga	0.77	1.77
Gemella	1.53	0.93
Bacteroides	0.01	2.28
Megasphaera	0.75	1.15
Klebsiella	0.15	1.72
Corynebacterium	0.19	1.66
Actinomyces	1.21	0.56
Turicibacter	0.00	1.44
Phocaeicola	0.02	1.15
Other	8.60	21.08

Data are presented as %
